# Supplementary material for: The Effects of Calculated Remnant-Like Particle Cholesterol on Incident Cardiovascular Disease: Insights from a General Chinese Population
Source: J Clin Med. 2021 Jul 30;10(15):3388. doi: 10.3390/jcm10153388 (PMC8348383; doi:10.3390/jcm10153388)
Supplement: Supplementary file 1 [file jcm-10-03388-s001.zip › jcm-1294973-supplementary.pdf]

Supplementary Materials

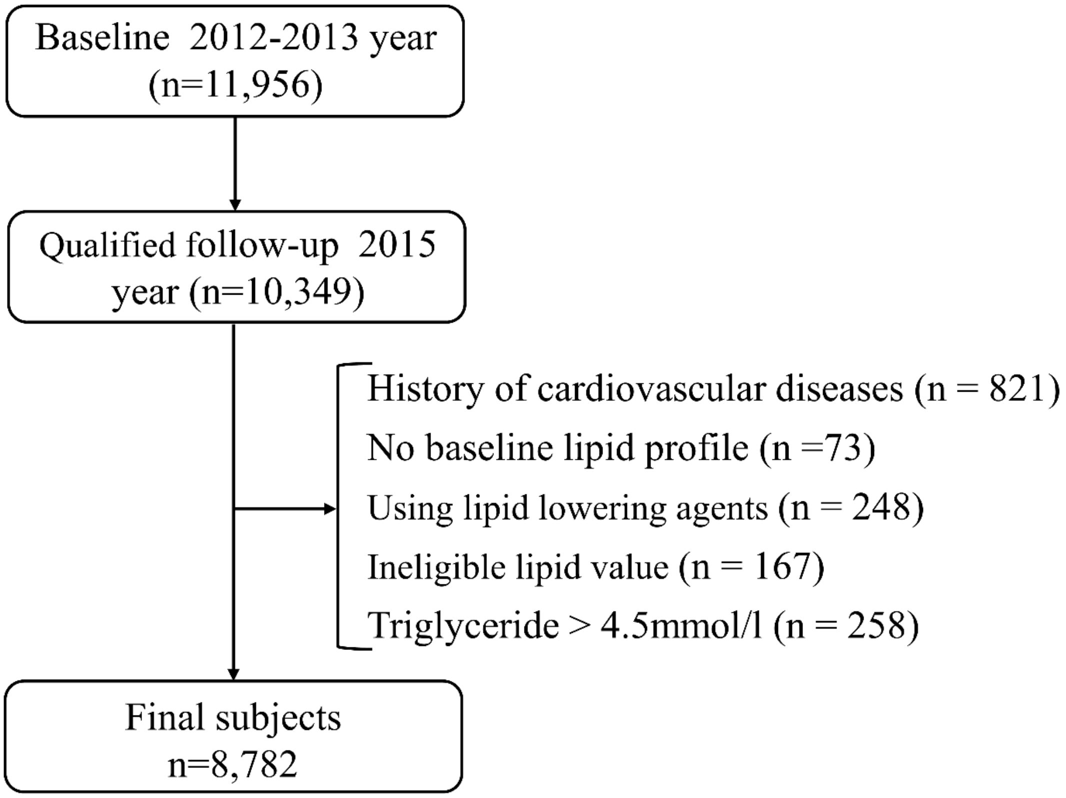

Supplementary Figure 1. Study flow chart

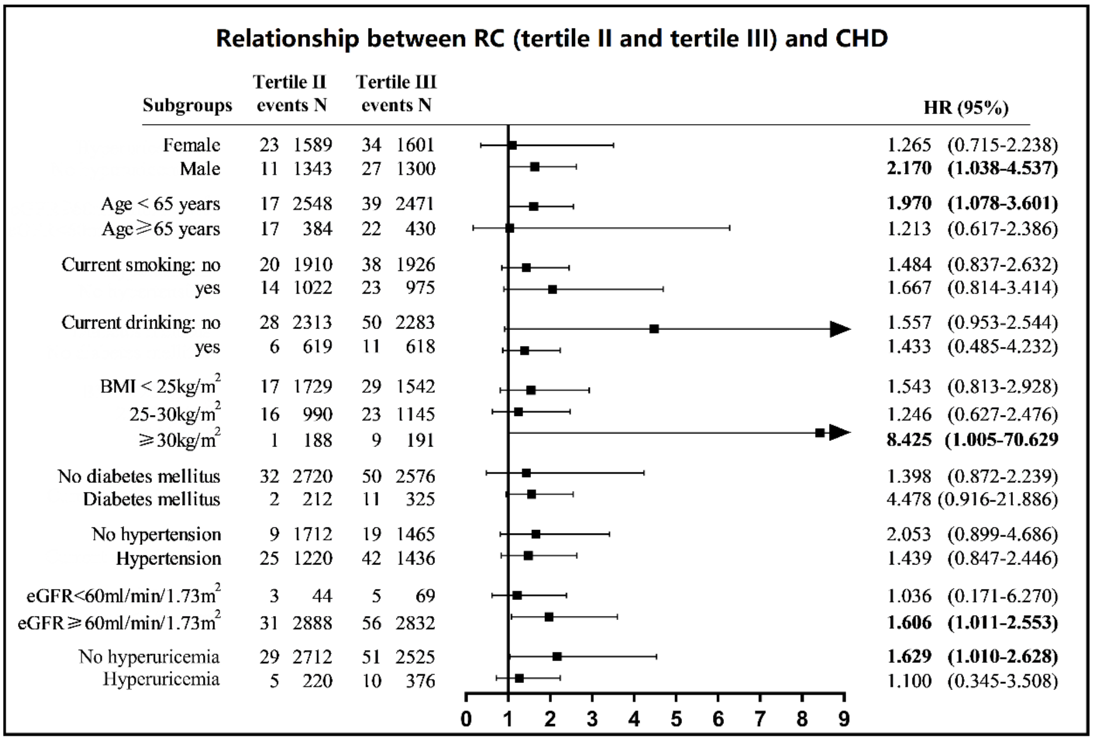

Supplementary Figure 2. Remnant cholesterol (tertile II and tertile III) in relation to coronary heart disease for different subgroups

Model adjusted for age, sex, ethnicity, body mass index, smoking status, drinking status, hypertension, diabetes mellitus, estimated glomerular filtration rate, hyperuricemia, and continuous triglyceride.

BMI, body mass index; CI, confidence interval; eGFR, estimated glomerular filtration rate; HR, hazard ratio.

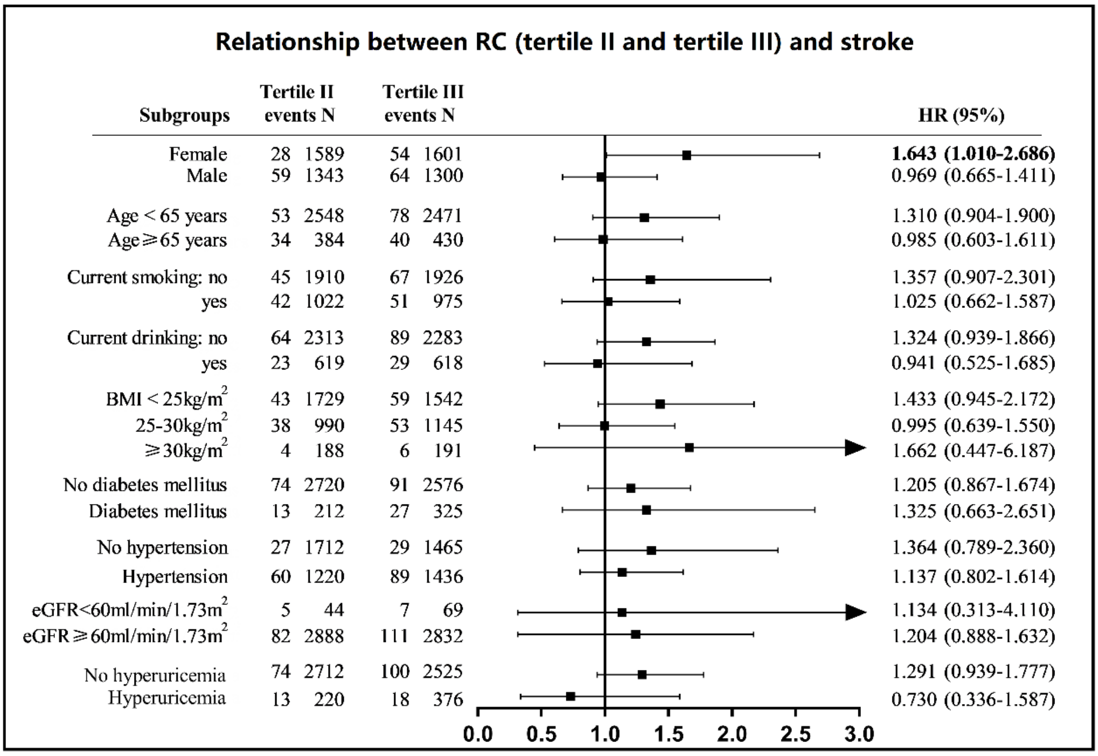

Supplementary Figure 3. Remnant cholesterol (tertile II and tertile III) in relation to stroke for different subgroups

Model adjusted for age, sex, ethnicity, body mass index, smoking status, drinking status, hypertension, diabetes mellitus, estimated glomerular filtration rate, hyperuricemia, and continuous triglyceride.

BMI, body mass index; CI, confidence interval; eGFR, estimated glomerular filtration rate; HR, hazard ratio.

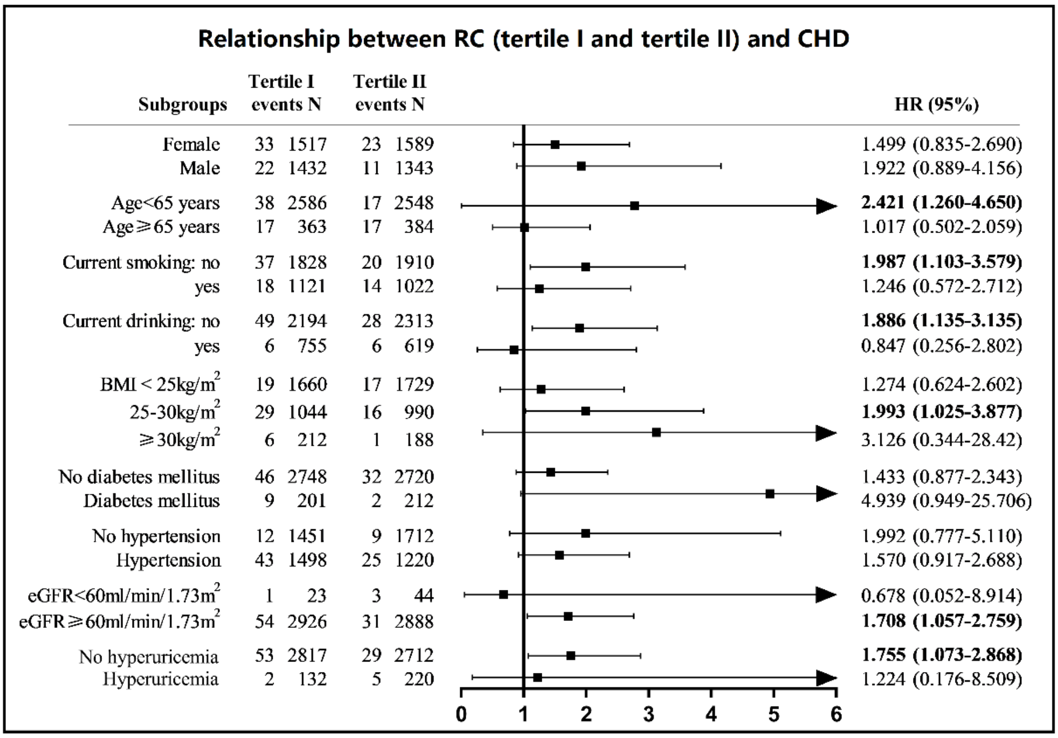

Supplementary Figure 4. Remnant cholesterol (tertile I and tertile II) in relation to coronary heart disease for different subgroups

Model adjusted for age, sex, ethnicity, body mass index, smoking status, drinking status, hypertension, diabetes mellitus, estimated glomerular filtration rate, hyperuricemia, and continuous triglyceride.

BMI, body mass index; CI, confidence interval; eGFR, estimated glomerular filtration rate; HR, hazard ratio.

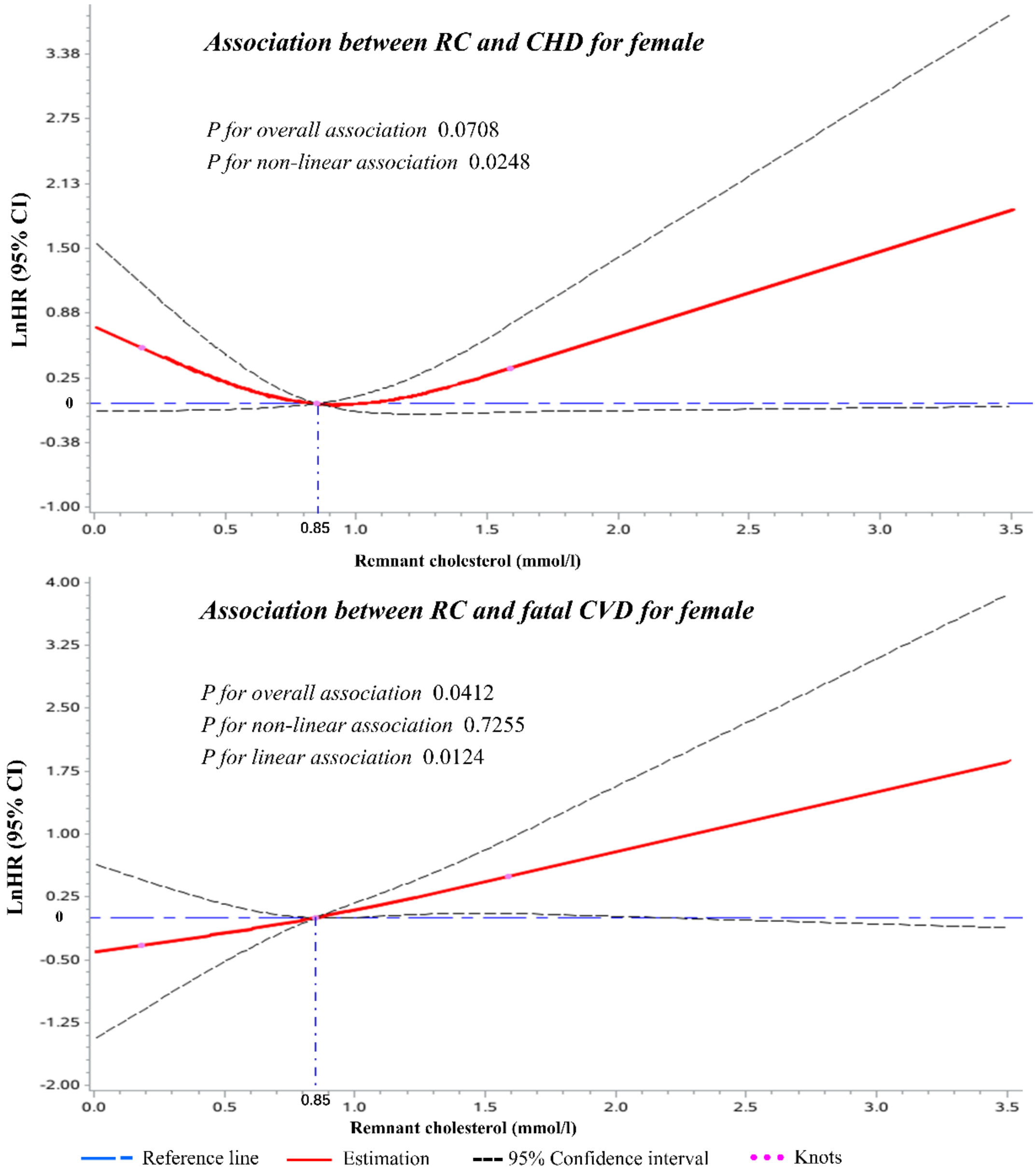

Supplementary Figure 5. Dose-response associations between RC and cardiovascular outcomes (CHD and fatal CVD) for female

Restricted cubic splines displaying the lnHRs of cardiovascular events with 95% confidence intervals according to serum level of remnant cholesterol. Reference set to medians (0.85mmol/l) level of remnant cholesterol for subgroup female. Knots located at 5th, 50th and 95th percentiles. Adjusted for age, sex, ethnicity, body mass index, smoking status, drinking status, hypertension, DM, estimated glomerular filtration rate, hyperuricemia, and continuous triglyceride. CHD, coronary heart disease; CVD, cardiovascular disease; HR, hazard ratio; lnHRs, natural log of hazard ratios; RC, remnant cholesterol; SD, standard deviation.

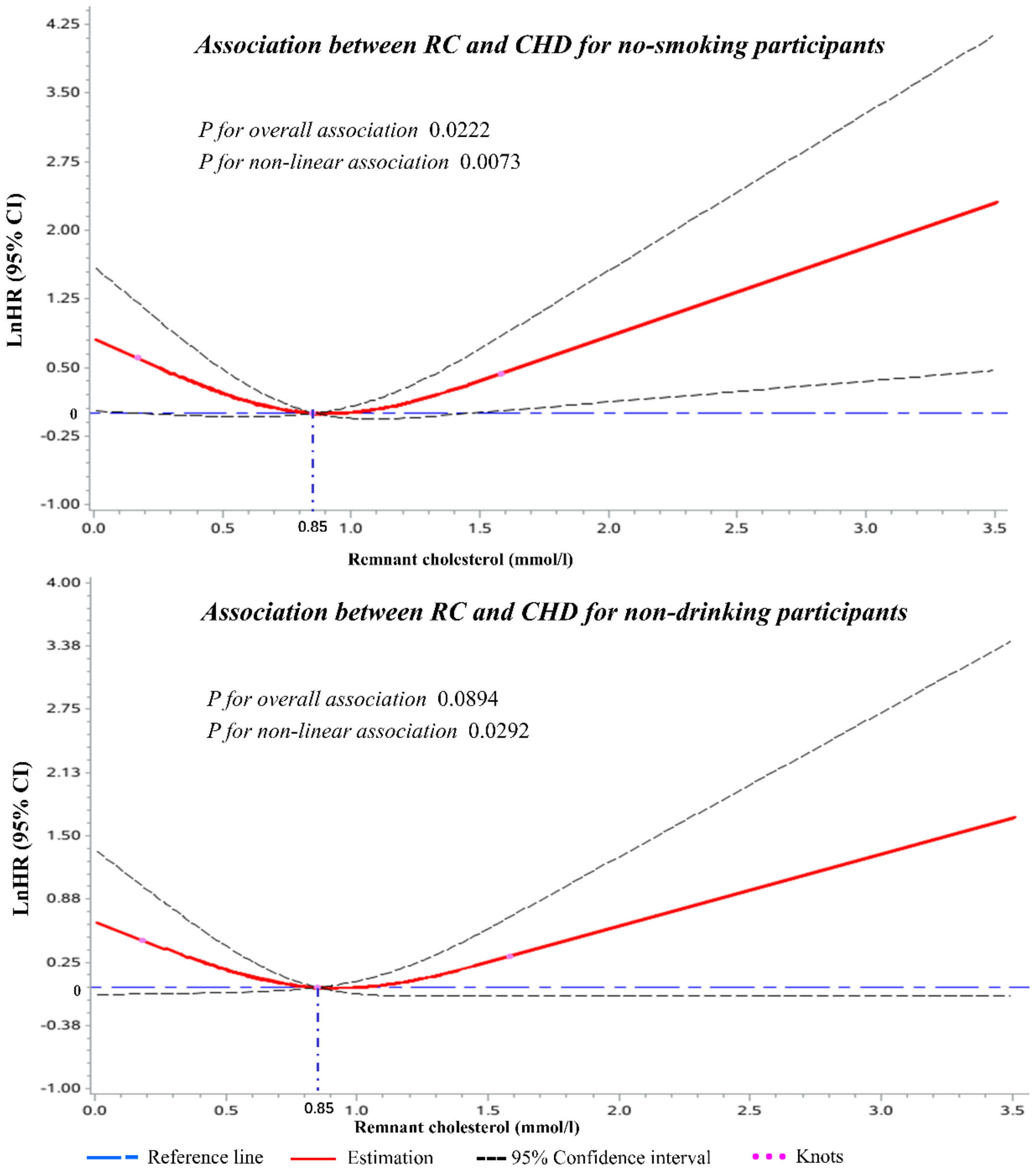

Supplementary Figure 6. Dose-response associations between RC and CHD for non-smokers and non-drinkers

Restricted cubic splines displaying the lnHRs of coronary heart disease with 95% confidence intervals according to serum level of remnant cholesterol. Reference set to medians (0.85mmol/l) level of remnant cholesterol for non-smokers and non-drinkers. Knots located at 5th, 50th and 95th percentiles. Adjusted for age, sex, ethnicity, body mass index, smoking status, drinking status, hypertension, DM, estimated glomerular filtration rate, hyperuricemia, and continuous triglyceride. CHD, coronary heart disease; HR, hazard ratio; lnHRs, natural log of hazard ratios; RC, remnant cholesterol; SD, standard deviation.

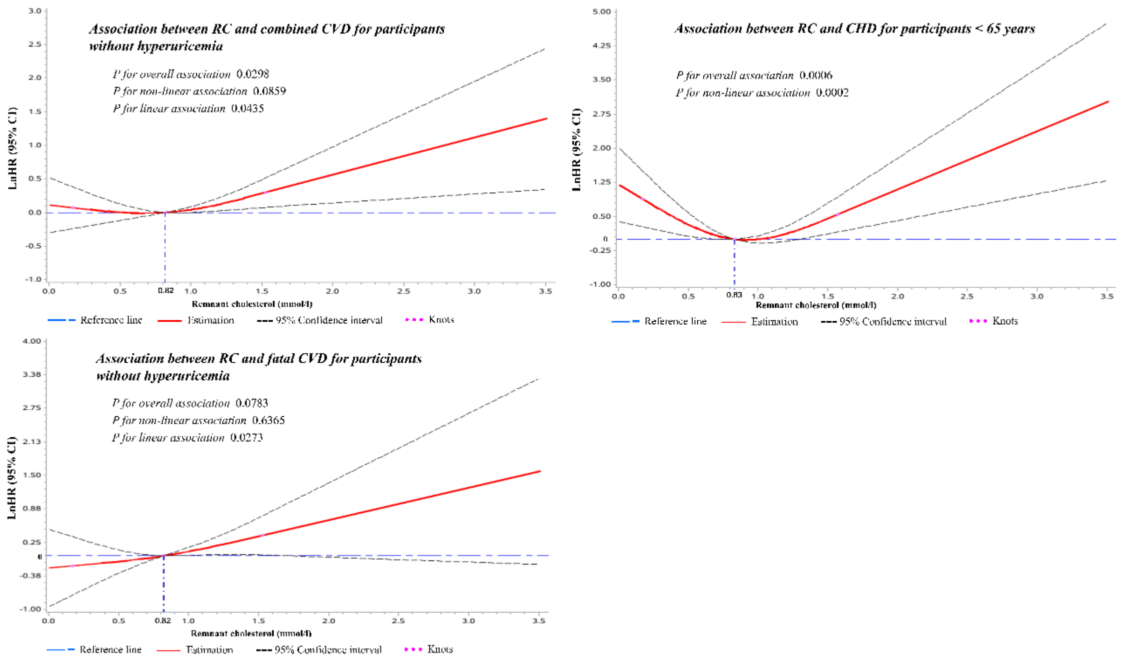

Supplementary Figure 7. Dose-response associations between RC and cardiovascular outcomes for participants without hyperuricemia

Restricted cubic splines displaying the lnHRs of cardiovascular outcomes with 95% confidence intervals according to serum level of remnant cholesterol. Reference set to medians (0.82mmol/l) level of remnant cholesterol for participants without hyperuricemia. Knots located at 5th, 50th and 95th percentiles. Adjusted for age, sex, ethnicity, body mass index, smoking status, drinking status, hypertension, DM, estimated glomerular filtration rate, hyperuricemia, and continuous triglyceride. CHD, coronary heart disease; CVD, cardiovascular disease; HR, hazard ratio; lnHRs, natural log of hazard ratios; RC, remnant cholesterol; SD, standard deviation.
